# Design and methods of a longitudinal study investigating the impact of antiretroviral treatment on the partnerships and sexual behaviour of HIV-infected individuals in rural KwaZulu-Natal, South Africa

**DOI:** 10.1186/1471-2458-11-121

**Published:** 2011-02-19

**Authors:** Nuala McGrath, Linda Richter, Marie-Louise Newell

**Affiliations:** 1 London School of Hygiene and Tropical Medicine, London, UK; 2 Africa Centre for Health and Population Studies, University of KwaZulu-Natal, Somkhele, South Africa; 3 Human Sciences Research Council, Durban, South Africa; 4 UCL, Institute of Child Health, London, UK

## Abstract

**Background:**

Diagnosed HIV-infected people form an increasingly large sub-population in South Africa, one that will continue to grow with widely promoted HIV testing and greater availability of antiretroviral therapy (ART). For HIV prevention and support, understanding the impact of long-term ART on family and sexual relationships is a health research priority. This includes improving the availability of longitudinal demographic and health data on HIV-infected individuals who have accessed ART services but who are not yet ART-eligible.

**Design and methods:**

The aim of the study is to investigate the impact of ART on family and partner relationships, and sexual behaviour of HIV-infected individuals accessing a public HIV treatment and care programme in rural South Africa. HIV-infected men and women aged 18 years or older attending three clinics are screened. Those people initiating ART because they meet the criteria of WHO stage 4 or CD4 ≤ 200 cells/μL are assigned to an 'ART initiator' group. A 'Monitoring' group is composed of people whose most recent CD4 count was >500 cells/μL and are therefore, not yet eligible for ART. During the four-year study, data on both groups is collected every 6 months during clinic visits, or where necessary by home visits or phone. Detailed information is collected on social, demographic and health characteristics including living arrangements, past and current partnerships, sexual behaviour, HIV testing and disclosure, stigma, self-efficacy, quality of family and partner relationships, fertility and fertility intentions, ART knowledge and attitudes, and gender norms. Recruitment for both groups started in January 2009. As of October 2010, 600 participants have been enrolled; 386 in the ART initiator group (141, 37% male) and 214 in the Monitoring group (31, 14% male). Recruitment remains open for the Monitoring group.

**Discussion:**

The data collected in this study will provide valuable information for measuring the impact of ART on sexual behaviour, and for the planning and delivery of appropriate interventions to promote family and partner support, and safe sexual behaviour for people living with HIV in this setting and elsewhere in sub-Saharan Africa.

## Background

Diagnosed HIV-infected people form an increasingly large sub-population in South Africa, one that will continue to grow with widely promoted HIV testing and greater availability of antiretroviral therapy (ART). Factors that have been identified as being associated with unsafe sexual behaviours in HIV-infected people include: age, income[1], depression[2], drug and alcohol use[3,4], low self-efficacy[5], attitudes to condoms[1] and suprainfection, conception[6], duration of relationship[7], intimate partner violence[8,9], perceived stigma, and disclosure to family and partner(s)[10-12]. The relative influence of risk factors may change over time in a population of HIV-infected people, for example, as a result of changes in sexual behaviour norms. The determinants of unsafe sexual behavior can also change over time for a HIV-infected person depending on factors including the length of time since HIV diagnosis, the quality of counselling and support, and the HIV status of their partner. Many HIV-infected people are in HIV discordant couples[13] for whom practicing safer sex consistently over a long period may be challenged by numerous factors including emotional and sexual intimacy issues, poor communication, and stresses including concerns about family disclosure and childbearing[2,14-16].

For HIV-infected people, experiences associated with morbidity and HIV treatment may have important influence on their family and partnering relationships, and sexual behaviours. ART reduces infectivity, however, it also increase the duration of potential infectiousness by reducing morbidity and mortality, and may also result in risk compensation[17]. Thus, efforts to promote safer sex (for example, reduction of concurrent partnerships, reduction of the number of partnerships, condom use, abstinence) in people receiving HIV treatment and their partners remains an important public health goal.

Several direct and indirect effects of ART have been identified on psycho-sexual functioning [18-20]. Side effects, in particular lipodystrophy, may result in sexual dysfunction[21]. Low self-efficacy is associated with poor adherence[22-24], as well as, a higher risk of unsafe sexual behaviours[25]. However, there is a pressing need to monitor sexual risk behaviours of HIV-infected people over time to measure the full extent of long-term ART on sexual behaviour and HIV transmission[26-28]. Studies in Europe, US and Africa have reported that HIV-infected individuals receiving ART do not have increased sexual risk behaviour[29-34]. Yet, in order to inform secondary prevention efforts, greater knowledge about family and partnering contexts, issues related to stigma and HIV disclosure, and characteristics associated with unsafe sexual behaviours of HIV-infected people is needed. This information is especially important in sub-Saharan Africa where the HIV epidemic has been most severe and public access to HIV treatment is relatively recent compared to developed countries.

A group of HIV-infected people little studied with respect to their sexual behavior are HIV-infected individuals who have accessed ART services but who are not yet ART-eligible. This group are difficult to prospectively study as they have high attrition from HIV clinics[35], and the period of follow-up after diagnosis of HIV may be short as individuals initiate ART or die. Nonetheless it is important to learn more about this group for two reasons. First, HIV prevention strategies may need to be specifically tailored for people who know their HIV positive status but do not yet require treatment. Second, this group provides an appropriate comparison for studying the impact of ART initiation on sexual behaviour.

In Africa, the few studies that have sought to measure the impact of ART initiation on sexual behavior vary widely in their design. In a study by Bunnell et al in Uganda, there was no comparison group with which to compare the observed changes in sexual behaviour in HIV-infected people receiving ART[34]. One South African study used two separate cross-sectional samples of HIV-infected people waiting to start ART to control for sexual behaviour trends in a cohort of people initiating ART[33]. A limitation of repeated samples is that each sample may differ in important ways that are unmeasured. Consequently, a lack of difference in sexual behaviours between the two cross-sectional samples may be an artifact of the sampling process. Other cohort studies in Africa have compared sexual behavior pre-ART with sexual behavior reported after ART initiation[31,32]. In these studies, the same individual may therefore contribute to pre- and post-ART exposure groups. A limitation of this approach is that for each individual, the pre-ART period will always be earlier than their post-ART period, and thus, any secular changes in sexual behaviours during the period of study might erroneously be interpreted as an impact of ART. Adjusting for time in the cohort[31] or conducting sensitivity analyses[32] provides information with which to assess whether study results may have been influenced by factors other than ART. In our study of long-term sexual behaviors of participants in a rural ART programme in South Africa, we used a different study design; that of prospectively observing sexual behavior of pre- and post-ART groups simultaneously. Our control group consists of people not yet eligible for ART who are followed longitudinally at the same time as a group starting ART.

## Aims and objectives

This study aims to investigate the impact of antiretroviral treatment (ART) on family and partner relationships, and sexual behaviour of HIV-infected individuals in rural South Africa by following a prospective cohort of HIV-infected individuals in the Hlabisa sub-district of Umkhanyakude in northern KwaZulu-Natal, South Africa. The sample consists of people initiating ART and those not yet eligible for ART (monitoring group). This is a joint study between the Africa Centre for Health and Population Studies which is part of the University of Kwazulu-Natal (UKZN), the South African Human Sciences Research Council (HSRC) and the London School of Hygiene and Tropical Medicine. The study is funded by the Wellcome Trust through a fellowship award to McGrath.

The specific objectives of this study are to

1. Describe sexual behaviour and partnership attitudes and partner change over a three year period among ART initiators and those being monitored for ART

2. Explore which clinical and psychosocial factors are associated with sexual behaviour change among ART initiators and those being monitored for ART

3. Explore how clinical and psychosocial factors change over time among ART initiators and those being monitored for ART

4. Study whether sexual behaviour changes as people transition from pre- and early to longer term treatment

## Target population

The target population is HIV-infected individuals who are resident in the Africa Centre Demographic Surveillance Area (ACDSA) in the rural Umkhanyakude district of KwaZulu-Natal, South Africa, and accessing ART clinics for HIV care. Since 2000, the Africa Centre Demographic Information System (ACDIS) has collected longitudinal social, demographic and health data from a Zulu speaking population of approximately 86,000[36] (see http://www.africacentre.ac.za). This population has experienced a severe HIV epidemic, with prevalence in 2003/4 peaking at 50.9%, 95%CI (47.2-54.6) among resident females aged 25-29 years and 43.5% (38.0-49.0) among resident males aged 30-34 years[37], and continued high crude HIV incidence rates per 100 person-years of 3.8 (95% CI, 3.2-4.6) among women aged 15-49 years and 2.3 (95% CI, 1.8-3.1) among men aged 15-54 years[38]. The study area has a decentralized programme of HIV treatment and care is delivered through a network of 17 primary health care clinics in the Hlabisa subdistrict of Umkhanyakude[39]; 6 of these clinics are located within the ACDSA. The programme, which started in late 2004, is a partnership between the local Department of Health and the Africa Centre for Health and Population Studies, and follows the South African Comprehensive HIV and AIDS Care, Management and Treatment Plan, (see Houlihan et al, 2010, for details of Programme procedures for HIV diagnosis, ART eligibility, treatment regimens and follow-up[40]). The Africa Centre has established research facilities alongside the government ART clinics.

Routine data collected at the clinics include information about ART initiation, ART combination prescribed, vital status, TB status and treatment, pregnancy, viral load and CD4. This information is entered into a database developed and maintained at the Africa Centre (ART Evaluation and Monitoring Information System, or ARTemis), and is updated with each repeated visit to the clinic. Laboratory results (CD4 cell count and HIV load) are also regularly imported into ARTemis from the laboratory at the local district hospital or from clinic records. The ACDIS database provides longitudinal demographic information including pregnancy histories for women, household living arrangements, mobility and socioeconomic status. It is possible to link individuals who are in ARTemis with the ACDIS databases if they are a member of a household in the ACDSA, through an individual's South African identity number[39].

## Methods/Design

HIV-infected men and women, accessing the HIV treatment and care programme at any of the three busiest ART clinics in the ACDIS surveillance area, who are aged 18 years or older and resident within the ACDSA are potentially eligible for this study. In addition, individuals must be willing to participate in a study interview at the clinic every 6 months; be willing to answer questions about themselves, their relationships and sexual activity; and be prepared to provide phone contact details to permit study staff to contact them for tracking and queries.

This study follows two groups over time (1) those starting ART (ART initiators) and (2) those who are not yet eligible for treatment (Monitoring group). Individuals who are eligible to initiate ART (i.e. WHO stage 4 or CD4 ≤ 200 cells/μL), are assigned to the ART initiator group. If individuals eligible for the study have a CD4 count >500 cells/μL from a recent CD4 test, they are assigned to the Monitoring group. A cut-off of CD4>500 cells/μL was chosen because we wished to collect repeated measures of this group over time before they initiate treatment, and prior studies suggest that the median time from presenting with a count between 500 and 650 cells/μL to having a count <350 cells/μL is 2.5 years, with approximately 75% and 90% having <350 cells/μL within 5 and 8 years, respectively[41]). Individuals with CD4 between 200 cells/μL and 500 cells/μL are excluded from the study because they are likely to start ART in the short-term and would contribute limited person-years of follow-up to the Monitoring group, particularly relevant in light of any changes in the South African programme guidelines for ART eligibility. Any individual who has previously received ART and defaulted or is currently established on ART (for ≥2 weeks), planning to leave the area in the next 12 months, or pregnant at the time of recruitment, are also excluded from the study. Pregnant women are excluded because sexual activity is often different during pregnancy and CD4 counts are known to decline during pregnancy[42], providing unsuitable baseline data. If a female participant in the study becomes pregnant during follow-up, they remain in the study.

## Sample size calculations

Separate sample sizes were calculated for the two groups. The primary outcomes of interest are changes in sexual behaviour that occur by 2 years of follow-up. Current condom use was used for these calculations as an available indicator of change in sexual behaviour. In 2005, 16% of women aged 15-49 in ACDIS reported current use of condoms. Using this estimate as a baseline, and wishing to detect an increase in condom use to 30% at 2 years, with 80% power and alpha level of 0.05, requires 142 women. Conservatively assuming that 20% would be lost to follow-up by 2 years, the enrolment target was 178 women for each group. Men's reported current use of condoms in 2005 was higher, with 30% reporting condom use at last sex. Wishing to detect an increase in condom use from 30% to 50% at 2 years, with 80% power and alpha level of 0.05, requires 96 men. Conservatively assuming 20% would be lost to follow-up by 2 years, the enrolment target was 120 men for each group. Thus, our sample size for each group was 300 (178+122), with a target of 40% men. A recruitment target of 100 of each group (ART initiators vs monitored) was set for each of the 3 study clinics The protocol was written to include a review of loss to follow-up of both groups before recruitment closed, and allowed for an increase in sample size, if needed, to maintain study power.

## Recruitment procedures

A general introduction to the study is given each morning by study staff in the waiting room at the clinic, explaining why the study is taking place, what participation entails, what the anticipated benefits are and answering any queries about the study. Leaflets describing the study are also handed out. Additional study introductions are targeted to patients presenting for CD4 testing and they are asked to present themselves to a study staff member again when they return to the clinic two weeks later for their CD4 results if they are interested in joining the study.

When an individual receives their CD4 result at the clinic and, if they indicate that they are interested in participating in the study, the study staff member allocates a study number which is used on all forms, screens the individual using the screening checklist and, if eligible, they are led through the study information sheet and informed consent process.

In an effort to reduce the length of the interview at each visit, participants are asked during the informed consent process to separately consent to their study data being linked to their routine clinical data in ARTemis, and the ACDIS data. If participants do not agree to the linking, enrolment into the study is unaffected, and additional questions are asked at a follow-up visit to collect these data.

Patients are only enrolled after they have given fully informed consent in writing. No financial remuneration is offered to participants. Once enrolled, a baseline questionnaire is administered in a private room by a study staff member. The questionnaire is conducted while the participant is waiting to see ART clinic staff. Patients usually have to wait at ART clinic for several hours and, based on previous experience of working in the clinic, we are able to make provisions to ensure the participant does not lose their place in the clinic queue while participating in the study. Non-study staff working in the clinic are familiar with the study purpose and procedures and help facilitate the study by making potential participants aware of the study and by directing participants to the study room on busy days.

Individuals who are initiating treatment have three counseling sessions at the clinic before they receive ART. These visits to the clinic provide additional opportunities to enroll eligible ART initiators if they are not ready to enroll on the day they receive their CD4 result.

## Follow-up procedures

Follow-up for both the ART initiator group and the Monitoring group is scheduled for every 6 months, for a maximum of 8 follow-up visits over the four-year study period. The ART initiator group is interviewed at their closest monthly visit for treatment collection, and the Monitoring group is interviewed during their 6 monthly visit for CD4 testing.

A month before each scheduled study visit, participants in the ART initiator group are reminded of the planned interview by study staff when they collect their treatment at the clinic. If the participant is not seen at the clinic, they are contacted by cell phone.

For the Monitoring group, at each study visit, an appointment is set for the next scheduled visit 6 months later and an appointment card is completed and taken home by the participant. A week before the appointment date, the study staff reminds the participant by phone of their need to test for CD4 again, even if they feel well, and of their related upcoming study visit.

## Tracking procedure

During the baseline interview, the contact details of the participant and at least one other person who is likely to know the whereabouts of the participant are recorded. Detailed directions to the participant's residence are taken, and the names of the school, church and shop nearest to their current residence are also noted. In addition, details of an alternative household, defined as place they might visit when they are not staying at their usual residence, are also taken. These are updated on each subsequent contact.

Every effort is made by study staff to minimize loss to follow-up. If an individual misses their study appointment, phone contact is attempted. Home visits are made to investigate the status of defaulters, if phone contact has not been possible, and consent for home visits has been given during the informed consent process. If the individual moves out of the area, or is too busy to come to the clinic because they are temporarily away or working, the study team tries to arrange an appointment at a place convenient for the participant, or administers the questionnaire by phone.

## Staffing

The research assistants administering the questionnaires have specialized training and experience in asking sensitive questions, probing for consistency and clarity, transitioning from one section to another and keeping interviewees engaged; they also have strong HIV and ART counseling skills. Study staff are trained to follow Africa Centre standards and guidelines to protect confidentiality. Clinic staff cooperate with the study by facilitating participants stepping out of the ART clinic queue for interview, informing potential participants of the study and directing them to our study staff member.

## Data collection instruments

### Baseline

The questionnaire sought detailed information on sociodemographic characteristics (age, sex, marital history); socio-economic circumstances (employment, income, receipt of grants, household assets); religion; HIV testing history and disclosure of HIV status; stigma; self-efficacy; partnership history and current sexual behaviour (including condom use, concurrency and multiple partnerships); fertility and fertility intentions with current main partner and the quality of that relationship including questions about communication, conflict, stability, identity and commitment; knowledge of and attitudes to ART; social capital and gender norms. Other gender-specific information is also collected, specifically, current use of contraception among women, and circumcision and vasectomy status among men. Where possible, questions that had previously been administered in ACDSA, in South Africa, or elsewhere in Africa were used (eg. questions about fertility intentions[43] and social capital[44,45]). In the case of psychological scales, we adapted scales to measure gender norms[46] and stigma[47], and the scale items were administered during interviews with 3 options for an answer: agree, no opinion, and disagree. Copies of the questionnaire are available by request from NM.

Interviews are conducted in Zulu, therefore all questionnaires and informed consent documents were translated into Zulu. Translation and back-translation procedures were used to ensure the equivalence of the meaning of questions in English and Zulu. The questionnaire for the most part contains closed questions, but there are some open questions as well. Questionnaires were piloted extensively during the development of the study using focus groups with Africa Centre staff, and revised in the light of feedback and comments

### Follow-up

The follow-up questionnaires collect identical information to the baseline except to explore whether the individual's circumstances or experiences have changed in the period since the last interview. For stigma, gender norms, fertility intentions with current main partner and the quality of that relationship, status observations rather than changes since last interview are collected. In addition, the follow-up questionnaire collects information on substance use (alcohol and substance use), health-seeking behaviour and ART adherence, disclosure of ART and side-effect questions for those who are on ART.

To aid follow-up interviews, a summary of previously reported key information is extracted from the database and used to prepare the form for the upcoming visit. This has assisted in further building rapport during follow-up interviews and has enhanced reliability of reports. Furthermore, the need for respondents to answer complex, inter-related questions that can be cross-checked by the interviewer immediately encourages frank responses. Although face-to-face interviews about socially sensitive information are always subject to some social desirability bias in reporting, the interviews are conducted to encourage openness in a non-judgmental confidential setting. That this is successful is indicated by the many participants who contact study staff between study visits, by phone and in person, particularly those who are attending the clinic monthly to collect their ART.

## Analysis plan

To assess factors associated with being sexually active and changes in sexual activity longitudinally, we will conduct participant-level analyses (i.e. the unit of analysis will be the enrolled participant). Multivariable logistic regression models will be used to identify factors associated with sexually activity at baseline, and compare sexual activity at baseline with follow-up. Attitudes, indicators of relationship quality and, individual characteristics will be examined as possible modifiers of sexual behaviour change over time. The extent to which these factors change with the expanding availability of ART or with treatment duration will also be investigated. ART side effects data will help to separate physical factors from psychosocial reasons for changes in sexual behaviour. Analyses will be stratified by gender and the interaction between gender and age will be explored.

We will also conduct multivariable sexual partner-level analyses (i.e. the unit of analysis will be a sexual partner of a study participant). Rates of unsafe sex will be compared using a Poisson regression model. Characteristics of the partnership will be investigated as potential modifiers of the unsafe sex rate. Multiple partners of the same study participant will be included as separate units of analysis, and repeated measures will be reflected in the covariance structure for the models.

Table 1 shows the primary outcomes of interest in the study. These outcomes were chosen because previous work has shown that they are associated with the efficiency of HIV transmission per contact and exposure of susceptible persons to infected persons, as noted in the table [48]. The two study groups will be compared with respect to each outcome in order to estimate the impact of ART on that outcome.

**Table 1:** Study Outcomes and their definitions

**Outcome (at 2 years)**^**1**^	**Definition of outcome**	**Factor influencing efficiency of transmission**	**Factor reflecting exposure of susceptible to infected persons**
Proportion of sexual acts involving a condom	Number of sex acts protected by a condom throughout divided by the number of sex acts in the last month^2^	√	

Condom use at last sex	At last sex, use of condom throughout	√	

Unsafe sex	Inconsistent condom use with a HIV-negative or unknown serostatus partner in the past 6 months	√	√

Sexually active	One or more sex acts in the last month		√

Frequency of sex acts within each partnership	The number of sex acts with each partner in the last month		√

Rates of acquisition of new sex partners	The number of new partnerships in the last 6 months divided by the person years of follow-up in the last 6 months		√

Rates of dissolution of existing partnerships	The number of partnerships ended in the last 6 months divided by the person years of follow-up in partnership in the last 6 months		√

Frequency of concurrent partnerships	Number of overlapping sexual partnerships in which sexual intercourse with one partner occurs between two acts of intercourse with another partner^3^		√

Abstinence	No sexual intercourse during the past 6 months		√

^1 ^Analyses of these outcomes will also be conducted at earlier time-points.

^2 ^Information is collected in the last month; however, if there was no sex in the last month, the questions are asked about the last 3 months; and if no sex in the last 3 months, the questions are asked about the last 6 months

^3 ^UNAIDS Reference Group on Estimates, Modelling, and Projections: Working Group on Measuring Concurrent Sexual Partnerships. HIV: consensus indicators are needed for concurrency. Lancet. 2010 Feb 20;375(9715):621-2.

## Community entry and research ethics approval

Africa Centre policy requires that all new research projects are introduced to the community by the Africa Centre's community liaison office. Our study was introduced to the Africa Centre's Community Advisory Board (AC CAB) for their input and approval on May 29^th ^2008. The AC CAB, which meets on a regular basis, consists of about 40 members nominated by the community. In addition, the study protocol was presented to the Medical Manager of Hlabisa Hospital on June 3rd, 2008, and to Hlabisa Hospital Management in their management meeting on June 9^th^, 2008. All components of the study (ref BF083/08), including the linking of ARTemis data to the population-based ACDIS data (ref E134/06) were given approval by the University of KwaZulu-Natal ethics committee. Approval for the study protocol and associated information sheets, consent forms and questionnaires was also received from the Office of the Principal Technical Advisor on Research, in the provincial Department of Health in Pietermaritzburg, and from the London School of Hygiene and Tropical Medicine ethics committee (ref 5413).

## Discussion

### Study Progress: Recruitment phase

Recruitment for both groups started in January 2009. As of October 2010, 600 individuals have been enrolled; 386 in the ART initiator group (141, 37% male) and 214 in the Monitoring group (31, 14% male); recruitment only remains open for the Monitoring group. As expected, recruitment among the Monitoring Group compared to the ART initiator group is slower. It appears that the majority of people presenting for CD4 counts at local clinics are doing so because they are sick and therefore are likely to have CD4 counts well below 500, the minimum eligibility criteria for the Monitoring group. In addition, the small number of men recruited to this group to date appears to reflect the experience of the ART programme more generally. In an analysis of all ART-naive adults presenting with CD4 >200 cells/mm3 between Jan 1st and Dec 31^st ^2007 in the Hlabisa HIV Treatment and Care Programme, 83% were female[35].

To date, all participants have given consent to link their data collected in this study with their routine ART data and ACDIS data. Only one individual has refused to consent to being visited at home. The baseline interviews take a median of 31 minutes (IQR: 25-38).

In April 2010, national ART programme eligibility criteria were modified to allow all HIV-infected individuals with TB (after they complete the intensive phase of TB treatment), and pregnant women who have CD4 < 350, to start ART, in addition to those who have a CD4 ≤ 200 or are WHO stage IV. These changes in the programme have not affected recruitment because the majority of the ART initiator group had been recruited prior to the introduction of these additional criteria, but these changes may increase the proportion of the Monitoring group who initiate ART during the study.

As part of the study design, a 'recent' CD4 test was defined as less than 21 days before enrollment. However, this was found to be unreasonably restrictive given that CD4 results are only available at the clinic 2 weeks after testing (CD4 testing is performed centrally at Hlabisa hospital for the ART programme), giving little time for potential participants to present for their CD4 results at the clinic and be enrolled. Thus, the definition of 'recent' was relaxed to allow enrollment within 8 weeks of a CD4 test.

### Study Progress: Follow-up phase

As of October 8^th^, 2010, 542 of the enrolled individuals (359 (66%) in ART initiator group) were expected to have completed their 6 m visit. Overall, 485 (89%) had been interviewed again with no difference according to group (p = 0.51). In both groups, women were more likely to have been re-interviewed than men (92% vs 84% in the ART initiator group and 92% vs 85% in the Monitoring group respectively), although the difference was only statistically significant among the ART initiator group (p = 0.018).

In our study, we have tried to retain participants in the study by interviewing them elsewhere or over the phone if they do not return to the clinic. Seventy-three percent (73%) of the 6-month interviews completed in the ART initiator group were conducted at the clinic, compared to 61% for the Monitoring group (p = 0.024). Among those in the ART initiator group who were not interviewed at the clinic for their 6-month study visit, 55% were interviewed by phone, 32% at home, 8% at work and 5% elsewhere. Among those in the Monitoring group who were not interviewed at the clinic for their 6-month study visit, 43% were interviewed by phone, 46% at home, 5% at work and 6% elsewhere. In contrast, among individuals presenting for the first time to the local programme with CD4 >500, 40% returned for another CD4 test within 13 months, with a median time to return of 232 days (IQR 154-313), and men were less likely to return[35]. This suggests that our approach of allowing follow-up interviews to be conducted by phone is helpful for study retention.

### Summary

This clinic-based study will allow us to follow longitudinally over 600 HIV-infected individuals receiving ART and/or care in rural KwaZulu-Natal. The data collected in this study will provide an opportunity to estimate the impact of ART on sexual behaviour. In addition, the study data will provide valuable information for the planning and delivery of appropriate interventions to promote family and partner support, and safe sexual behaviour for people living with HIV not only in rural KwaZulu-Natal but in other parts of sub-Saharan Africa.

## Competing interests

The authors declare that they have no competing interests.

## Authors' contributions

NM led study design and writing. All authors contributed to study design and drafting the manuscript. All read and approved the final manuscript.

## Funding

The HIV treatment and care programme has received generous support from the United States Agency for International Development (USAID) and the President's Emergency Plan for AIDS Relief (PEPFAR) under the terms of award no. 674-A-00-08-0001-00. The opinions expressed herein are those of the authors and do not necessarily reflect the views of USAID or the United States Government. The Africa Centre is supported by a core grant from the Wellcome Trust. Nuala McGrath was supported by a Wellcome Trust fellowship (grant # WT083495MA).

## Pre-publication history

The pre-publication history for this paper can be accessed here:

http://www.biomedcentral.com/1471-2458/11/121/prepub
